# Glucose Metabolic Brain Network Differences between Chinese Patients with Lewy Body Dementia and Healthy Control

**DOI:** 10.1155/2018/8420658

**Published:** 2018-05-08

**Authors:** Danyan Chen, Jiaying Lu, Hucheng Zhou, Jiehui Jiang, Ping Wu, Qihao Guo, Jingjie Ge, Huiwei Zhang, Kuangyu Shi, Chuantao Zuo

**Affiliations:** ^1^Shanghai Institute for Advanced Communication and Data Science, Shanghai University, Shanghai, China; ^2^PET Center, Huashan Hospital, Fudan University, Shanghai, China; ^3^Institute of Biomedical Engineering, Shanghai University, Shanghai, China; ^4^Department of Neurology, Huashan Hospital, Fudan University, Shanghai, China; ^5^Department of Nuclear Medicine, Technische Universität München, Munich, Germany; ^6^Institute of Functional and Molecular Medical Imaging, Fudan University, Shanghai, China

## Abstract

Dementia with Lewy bodies (DLB) is the second most common degenerative dementia of the central nervous system. The technique ^18^F-fluorodeoxyglucose positron emission tomography (^18^F FDG PET) was used to investigate brain metabolism patterns in DLB patients. Conventional statistical methods did not consider intern metabolism transforming connections between various brain regions; therefore, most physicians do not understand the underlying neuropathology of DLB patients. In this study, ^18^F FDG-PET images and graph-theoretical methods were used to investigate alterations in whole-brain intrinsic functional connectivity in a Chinese DLB group and healthy control (HC) group. This experimental study was performed on 22 DLB patients and 22 HC subjects in Huashan Hospital, Shanghai, China. Experimental results indicate that compared with the HC group, the DLB group has severely impaired small-world network. Compared to those of the HC group, the clustering coefficients of the DLB group were higher and characteristic path lengths were longer, and in terms of global efficiencies, those of the DLB group was also lower. Moreover, four significantly altered regions were observed in the DLB group: Inferior frontal gyrus, opercular part (IFG.R), olfactory cortex (OLF.R), hippocampus (HIP.R), and fusiform gyrus (FFG.L). Amongst them, in the DLB group, betweenness centrality became strong in OLF.R, HIP.R, and FFG.L, whereas betweenness centrality became weaker in IFG.R. Finally, IFGoperc.R was selected as a seed and a voxel-wise correlation analysis was performed. Compared to the HC group, the DLB group showed several regions of strengthened connection with IFGoperc.R; these regions were located in the prefrontal cortex and regions of weakened connection were located in the occipital cortex. The results of this paper may help physicians to better understand and characterize DLB patients.

## 1. Introduction

Dementia with Lewy bodies (DLB) is the second most common degenerative dementia of the central nervous system. Its clinical symptoms include fluctuating levels of cognition, cognitive impairment, parkinsonism, and visual hallucination [1]. Because clinical, neuropsychological, and pathological features of DLB are similar to those of Parkinson's disease (PD) and other dementia subtypes, such as Alzheimer's disease (AD), the rate of misdiagnosis has increased despite the publication of refined clinical criteria for DLB [2]. In clinical practice, it is necessary to have an exact diagnosis and identification of DLB.

In recent years, in order to better understand and characterize DLB patients, few scholars have used functional imaging techniques to elucidate brain metabolism patterns in DLB patients. Amongst them, ^18^F fluorodeoxyglucose positron emission tomography (^18^F- FDG PET) is considered as the gold standard [3] due to its high accuracy. Recently, ^18^F-FDG PET imaging has been used to identify different disease-specific patterns [4] that disrupt metabolic connectivity (MC). Moreover, it is being increasingly used in routine clinical practice [5].

Many research studies have described the advantages of ^18^F-FDG PET technique. O'Brien et al. conducted an experimental research study to compare the accuracy with which ^18^F-FDG PET and (hexamethylpropyleneamine oxime (HMPAO)) SPECT cerebral perfusion could diagnose DLB. They found that both modalities showed broadly similar patterns: parietal and temporal lobes of dementia patients showed reduced levels of activity. Moreover, DLB patients showed reduced uptake in the occipital lobe. Nevertheless, ^18^F-FDG PET was significantly superior to cerebral blood flow SPECT [6]. There is consistent evidence to prove that DLB patients develop a specific dysfunctional pattern, characterized by significant hypometabolism in the occipital and parietotemporal lobes. In the frontal cortex of DLB patients, metabolism is reduced to a lesser extent [2]. For instance, DLB patients showed posterior brain hypometabolism primarily involving parietooccipital regions [7]. In particular, cingulate island sign (CIS), which refers to sparing of the posterior cingulate relative to the precuneus and cuneus, has been proposed as an FDG-PET imaging biomarker of DLB [8].

Although some initial findings can be found in previous research, more evidences are needed to validate these findings. As a complex integrative system in which billions of neurons are connected with each other, the human brain continuously processes and transmits information between spatially distributed but functionally linked regions [9]. However, such connections between functionally linked regions are not considered in traditional statistical methods. Therefore, scholars seed a new method to optimize current findings.

Brain network analysis may be a good alternative for addressing such issues. Presently, abnormal topology of different diseases can be determined with this method. Thus, the pathological mechanism of different diseases, such as Alzheimer's disease or schizophrenia, may be understood by a new perspective. Thus, it is possible to have an early diagnosis and evaluation of diseases with brain network imaging biomarkers [10]. For instance, using resting-state functional magnetic resonance imaging (rs-fMRI), researchers performed brain network analysis to assess brain network organization in patients diagnosed with DLB (22 patients) and Alzheimer's disease (24 patients). They found that global brain network measures of DLB patients were significantly different from those of Alzheimer's patients and healthy controls. By studying the metabolic network of DLB patients, researchers gained important insights into the link between local vulnerabilities, long-range disconnection, and neuropathological differences. For instance, Caminiti et al. studied fMRI images of 42 DLB patients and 42 healthy controls (HC) using sparse inverse covariance estimation method and graph theory. These methods revealed substantial alterations in connectivity indexes, brain modularity, and hub configuration. Compared to healthy controls, local metabolic connectivity was significantly decreased within the occipital cortex, thalamus, and cerebellum of DLB patients. However, local metabolic connectivity was significantly increased in the frontal, temporal, parietal, and basal ganglia regions of DLB patients. There were also long-range disconnections between these brain regions. This implies that the functional hierarchy of a normal brain was disrupted in DLB patients [11]. However, these studies were mainly conducted on Western subjects. These results may not be applicable to Chinese subjects.

The two main objectives of this study are therefore as follows: (1) to explore the differences in glucose metabolism of Chinese patients with DLB and to compare related parameters with HC and (2) to identify altered hubs of brain networks and to locate regions significantly correlated with altered hubs of brain networks in DLB patients. Finally, clear evidences on the differences between ^18^F-FDG PET images of DLB and HC subjects are expected to be presented from the aspect of brain connectome.

## 2. Experimental Procedure

### 2.1. Subjects

In this study, the experimental data were obtained from the PET Center of Huashan Hospital in Shanghai, China. All participants were right-handed, including 22 normal subjects and 22 DLB patients. Standardized uptake value ratios (SUVR) of FDG were calculated by dividing the cerebellar cortex-standardized uptake values. Three days before or after PET image acquisition, we obtained basic information of these subjects, including age, gender, and Mini-Mental State Examination (MMSE) values.  Table 1 displays statistical information of all participants.

### 2.2. PET Image Acquisition and Preprocessing

Whole brain PET images of 44 participants were acquired using Siemens Biograph 64 PET/CT machine, which was present in the PET Center of Huashan Hospital in Shanghai, China. The spatial resolution of the PET scanner was 5.9 mm full width at half maximum (FWHM) in the transaxial plane and 5.5 mm FWHM in axial plane. All subjects were intravenously injected with 185 MBq FDG in a dimly lit, quiet room. They were asked to keep their eyes closed for 60 min in order to minimize the confounding effects of any activity. Thereafter, static emission scans were continued for 10 min. Using a Shepp–Logan filter, we implemented a filtered back projection algorithm to reconstruct transaxial images of the following dimensions: 168 × 168 × 148 matrices and a size of 2.0 × 2.0 × 1.5 mm. The institutional review board of Huashan Hospital approved the acquisition of PET images [11]. The informed consent form was signed by all participants of this study.

All the original images were obtained in Digital Imaging and Communications in Medicine (DICOM) format. They were converted to NIfTI format using DCM2NII software (https://www.nitrc.org/projects/dcm2nii/). For preprocessing converted images, Statistical Parametric Mapping 8 (SPM8) software was implemented in MATLAB 2014a. Firstly, PET images were spatially normalized to the Montreal Neurological Institute (MNI, McGill University, Montreal, Canada) space. In this step, individual images were spatially warped to a reference PET template using SPM software. Spatial warping is a completely automated procedure based on 12-parameter affine transformation. Then, normalized images were smoothed by convolution using an isotropic Gaussian kernel with 10 × 10 × 10 mm^∗^3 FWHM. Finally, these images were transformed into gray level images with a grayscale of [0,255].

### 2.3. Brain Network Construction

**Figure 1:**
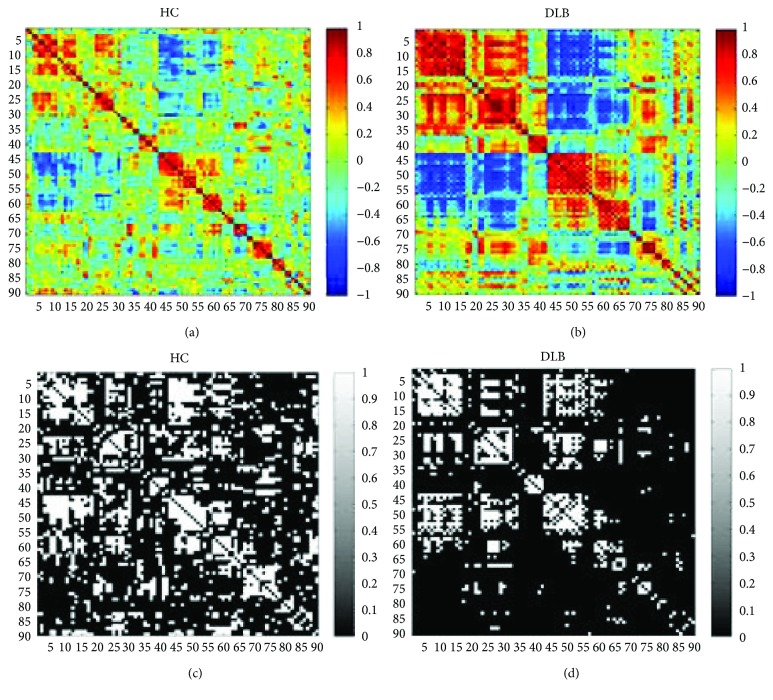
The partial correlation coefficient matrices and the binary matrices of the two groups. (a) The partial correlation coefficient matrix of the HC group. (b) The partial correlation coefficient matrix of the DLB group. (c) The binary matrix of the HC group in sparsity 21%. (d) The binary matrix of the DLB group in sparsity 21%.

Two networks of the HC and DLB groups were built based on two datasets, based on the definition of the network in graph theory [12]. The standardized AAL template contains 90 brain regions and 45 hemispheres, all of which are used to divide brain regions [13]. This AAL template was used to split all FDG-PET images into 90 nodes; each node implied a brain region. To further calculate the value of the node in the network, the average value of the intensity of each brain area was estimated. Then, we considered the value of each node as a whole and normalized it as a whole. Each node was normalized to zero-mean and unit-variance. It was obtained by subtracting the average value of the individual's whole brain and then dividing it by the standard deviation of the individual's whole brain. In previous studies, the partial correlation coefficient between each group of nodes was calculated, generating a partial correlation matrix of (90 × 90) samples. Figures 1(a) and 1(b) illustrate the partial correlation matrix of the HC and DLB groups, respectively.

In this study, a sparse threshold method was used [12]. The covariance correlation matrix represents an undirected weighted graph. Then, the partial correlation coefficient values of each two brain regions were calculated, which represented the connection strengths of the two brain regions. A larger partial correlation coefficient value indicated a stronger correlation between these two brain regions, which meant that the transform of glucose metabolism in these brain regions was more obvious. Furthermore, in this study, a binary comparison-weighted graph was used. A strategy was used to get the binary matrix and to set the sparsity of the connection matrix. In particular, the data was sorted in the connection matrix in terms of absolute value. Then, the sparsity was set to 21%, implying that the first 21% of the sequence was converted to 1. This indicates that a connection exists between two regions, vice versa, behind which the value would be converted to 0. This implies that there was no connection. With increasing sparsity, the connection matrix becomes sparser. Conversely, the greater the sparsity, the higher would be the density of the connected graph. Network topology is significantly impacted by the choice of sparse threshold. Instead of selecting a single sparse value randomly and subjectively, sparse values from sparsity (min = 0.06) to sparsity (max = 0.40) are considered. The two groups of subjects are connected with the lower limit. The choice of cap satisfies the strongly suppressed contribution of pseudocorrelation, ensuring that the resulting graph has a small world. What needs to be clarified is the fact that in the academic community, it is difficult to deal with negative correlations at the time of binarization. In our experiment, the negative correlation was converted to 1 once the absolute value of the negative correlation was greater than sparsity 21% [10].

Subsequently, the binarized network matrix can be generated. As shown in Figures 1(c) and 1(d), an element of 1 and 0 stands for “connection” and “no connection” in the binary matrix.

### 2.4. Network Parameter Analysis

To further investigate the differences between two sets of network parameters, the following parameters were considered: clustering coefficient (*C*), characteristic path length (*L*), local E, global E, *γ*, lambda, sigma, and node agent center (BC). All parameters were obtained from open-source toolkit GRETNA (https://www.nitrc.org/projects/gretna/) [14] and Brain Connectivity Toolkit (BCT, http://www.nitrc.org/projects/bct/) [15].

Based on graph theory, clustering coefficient *C* (*i*) was defined as the network that expressed a local connection probability, that is, the probability of connecting any two nodes around the node *i* [16]. The clustering coefficient *C* of the entire network was defined as the average of clustering coefficients of all nodes in the network, and it was a measure of the degree to which nodes in the graph tend to be clustered together [17]. In topology, the shortest path length was defined as the shortest path distance from node *i* to node *j*. In practice, it represents the minimum number of points connecting node *i* with node *j*. The shortest path lengths of all the nodes was calculated. The characteristic path length *L* was defined as the average of these path lengths. A network was formed with high global network efficiency [18]. If there was no connection between two points, then, path lengths between them was infinite.

If the network satisfies the clustering coefficient, then, it also satisfies the corresponding random network clustering coefficient ratio, *γ* = *C*/*C*_*r*. Moreover, it was determined using the characteristic path length; the clustering coefficient ≫ 1 satisfies the characteristic path length and the corresponding stochastic network. The average path length was greater than *λ* = *L*/*L*_rand = 1. For the comprehensive parameter *σ* = *γ*/*λ* > 1, it can be explained that the network had all the characteristics of a small world [16]. Random networks were obtained by randomly reattaching the original brain network between node edges. Meanwhile, the same number of nodes, edges, and degrees was preserved. To achieve statistical significances, we repeated 200 times random networks.

Global efficiency (global E) and local sales (local E) were also worked out to intuitively express transmission efficiency of the network at global and local levels. Global E is the average of shortest paths between nodes, reflecting the efficiency of the global network [19]. Local E is the average of the shortest paths between different connecting nodes, reflecting the efficiency of information exchange between subgraphs [20].

To further explore the nodes of this network, the concept of betweenness centrality was used. Betweenness centrality Bi of node *i* was defined as the number of shortest paths from all vertices to all others passing through node *i*. The betweenness centrality of the entire network was defined as the average of Bi from each node. In general, the Bi value was standardized while the normalized betweenness centrality of node *i* was defined by the expression: bi = Bi/*B*. From this definition, it can be inferred that the bi value was influenced by node *i* that transmits information in the entire network. Higher the bi value, stronger would be the regional centrality of the node. According to Seo et al. [21], hub regions of brain networks were proposed as nodes who have high bi values (bi > 1.5). To identify the hub region significantly altered, the results of the DLB group were compared with those of the HC group. A nonparametric permutation test was performed 1000 times. Statistical results were used to identify altered hubs (*p* < 0.05).  Table 2 summarizes all the parameters defined in this study.

### 2.5. Seed Correlation Analysis

Once altered hubs were determined for each of the two groups, they were considered as seed points and the relationship between them and rest voxels of the individual's brain was calculated. This step was to validate the significances of altered hubs. Pearson correlation coefficient method was used firstly. Then, Fisher's *r*-to-*z* transformation was used to convert Pearson's correlation coefficients to *z* values. Thus, an approximated Gaussian distribution was calculated using the following formula:

(1) $$\begin{array}{c} z_{i}=\frac{1}{2}\times \log\left[\frac{1+r_{i}}{1-r_{i}}\right]. \end{array}$$

Here, *r*_*i*_ refers to correlation coefficients and *z*_*i*_ refers to the transformed *z* value. Finally, *Z* test was used to compare *z* values between groups, whose expression is mentioned as follows:

(2) $$\begin{array}{c} z=\frac{z_{1}-z_{2}}{\sqrt{\left(1/\left(n_{1}-3\right)\right)+\left(1/\left(n_{2}-3\right)\right)}}. \end{array}$$

Here, *n*_1_ and *n*_2_ refer to samples of two groups [22]. To adjust variations associated with multiple comparison, a false discovery rate (FDR) method was performed at *q* value of 0.05.

### 2.6. Statistical Analysis

To confirm statistical significance of network parameters representing the DLB group and HC group, 2000 nonparametric permutation tests were performed [22].

To determine if the observed subject difference occurred accidentally (null hypothesis), we conducted further statistical analysis. The PET images of 44 participants were randomly assigned to the HC and DLB groups and then, the partial correlation matrix was calculated again according to Section 2.3. A set of binary matrices was obtained. The network parameters were also calculated for each network separately. The above process was repeated 1000 times, and the 95 percentile score for each differential distribution was considered as a cutoff (*p* < 0.05, one tailed). This meant that when the original results were within the first 5% of the random results, our results were considered to have significant differences.

## 3. Results

### 3.1. Network Parameters

As shown in Figures 1(a) and 1(b), partial correlation coefficient matrices of four groups were calculated using partial correlation analysis.  Figure 1 illustrates that color distribution was not uniform for different groups; therefore, partial correlation coefficient was inconsistent. The DLB group exhibited a deep colored distribution; its partial correlation coefficient had the highest absolute value. As shown in Figures 1(c) and 1(d), the threshold for binary matrices of two groups was at a fixed sparsity of 21% (refer to Section 3.2 for threshold selection).

**Figure 2:**
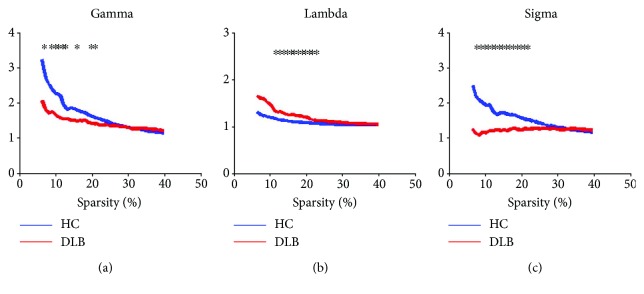
Gamma, lambda, and sigma values in the two groups. The asterisk refer to significant differences between the HC and DLB groups at the sparsity threshold (*p* < 0.05).

Figure 2 illustrates network parameters of gamma, lambda, and sigma for the DLB group and HC group. As shown in Figure 2, the entire threshold was ranging 6–40%. For both the DLB and HC groups, network parameters were as follows: gamma ≫ 1, lambda ≈ 1, and sigma > 1. A small-world network was observed in this study. Compared to the DLB group, this property was more obvious and noticeable in the HC group. At sparsity 18%, the value of sigma was 1.625 and 1.244 for the HC group and DLB group, respectively. By performing further permutation tests, it was found that over the entire threshold range, the gamma and sigma values in the DLB group were significantly smaller than those in the HC group. On the other hand, the lambda value was higher in the DLB group than in the HC group (*p* < 0.05).

**Figure 3:**
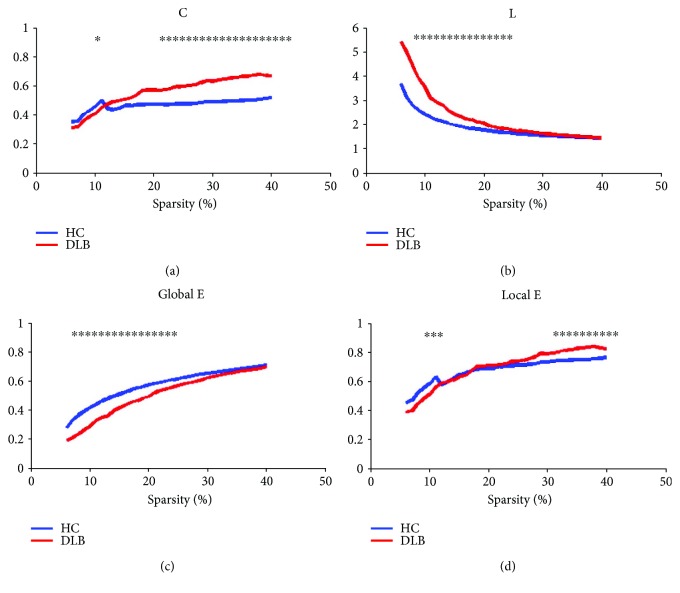
*C*, *L*, local E, and global E values in the two groups. The asterisk refer to significant differences between the HC and DLB groups at the sparsity threshold (*p* < 0.05).

As shown in Figure 3, network parameters *C*, *L*, global E, and local E were completely different for both the two groups. To compare the DLB group with the HC group, a nonparametric permutation test was performed to display statistical significance of differences between the two groups (*p* = 0.05).

### 3.2. Hub Regions

**Figure 4:**
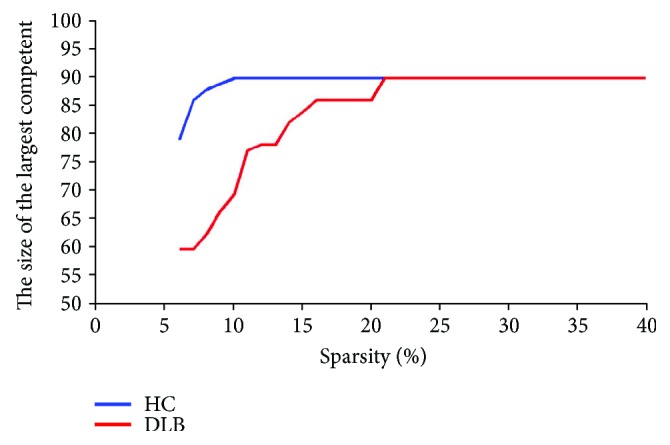
Different component of sizes of the two networks.

In a complex network, the normalized betweenness centrality (bi) was an important indicator of regional characteristics. These characteristics were used to determine the relative importance of network nodes and to identify key nodes in the network. These key nodes are defined as hubs in this study. To identify hubs in two groups, bi was calculated at a fixed sparsity of 21%. At the lowest sparsity of 21%, the component size was largest at 90. To guarantee >50 component size of the two networks, minimum sparsity thresholds were found to be 9% and 21% for the HC and DLB groups, respectively. At these thresholds, no node could be isolated from the remaining network.  Figure 4 shows different component sizes of two networks with a different sparsity threshold. As a result, sparsity 21% was chosen as the threshold and bi values were calculated for each node in the two networks [21].

**Figure 5:**
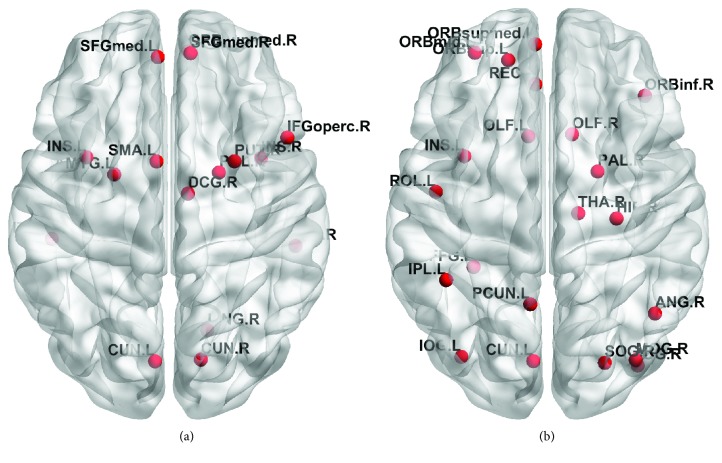
Hub nodes of two groups. (a) 16 hub nodes in the HC group. (b) 21 hub nodes in the DLB group.

At sparsity of 21%, 16 hubs and 21 hubs were identified for the HC and DLB groups, respectively. bi values and functional classification of hub regions were also presented in Tables 3 and 4, respectively. Functionally, hubs were primarily located in association areas for HC and DLB groups.  Figure 5 illustrates the results for hub nodes in the axial view. In the HC group, hubs are primarily located in the prefrontal and occipital cortices. In the DLB group, hubs are located in prefrontal, occipital, and subcortical cortices of the brain. (Figure 5 was drawn by BrainNet Viewer package [23]).

For comparing different groups, statistical analysis was done with permutation test. It was found that the DLB group showed significantly altered regions in the four brain regions (*p* < 0.05), including pars opercularis (IFGoperc.R), right lingual gyrus (LING.R), fusiform gyrus (FFG.L), and olfactory cortex (OLF.R). Amongst them, betweenness centrality in the DLB group became strong in OLF.R, HIP.R, and FFG.L compared to that in the HC group, whereas betweenness centrality became weak in IFG.R.  Table 5 shows betweenness centrality values and *p* values of these four hubs.

### 3.3. Seed Correlation

**Figure 6:**
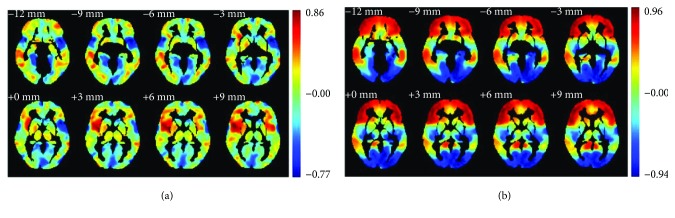
Seed correlation associated with the IFGoperc.R seed. (a) Correlation coefficient map with IFGoperc.R in the HC group; (b) correlation coefficient map with IFGoperc.R in the DLB group.

To further investigate connectivity between hubs in two groups, the right inferior frontal gyrus and pars opercularis (IFGoperc.R) were selected as the seed. The region was selected because of two primary reasons: IFGoperc.R is the hub's node in the HC group (bi > 1.5), but it is not targeted for hubs in DLB groups. The values of normalized bi were 1.83 and 0.08 in the HC group and DLB group, which has the smallest bi value in the DLB among hubs in the HC group. Compared with the HC group, IFGoperc.R status was different for DLB groups. Second, statistical analysis was conducted by conducting permutation test; it shows that for IFGoperc.R, bi values of DLB groups were significantly different from those of the HC group (*p* value = 0.0295). As shown in Figure 6, the results of voxel-wise correlation analysis were obtained with IFGoperc.R seed. Figure 6 illustrates the correlation coefficient map (R-map) associated with IFGoperc.R seed in the HC and DLB groups. (Figure 6 was drawn by the REST Slice Viewer [24]). In the HC group, the R-map shows that areas, which present positive connections with IFGoperc.R seed, were focused on the following components of parietal cortex: the bilateral postcentral gyrus (PoCG.R and PoCG.L), right middle frontal gyrus (MFG.R), and right angular gyrus (ANG.R). The weakened regions of the brain were mainly located in the following areas: the right and left cuneus (CUN.R and CUN.L), left insula (INS.L), and right parahippocampal gyrus (PHG.R).

In the DLB group, the prefrontal cortex showed enhanced brain activity in the following areas: the bilateral middle frontal gyrus (MFG.R and MFG.L), bilateral inferior frontal gyrus, pars triangularis (IFGtriang.R and IFGtriang.L), bilateral inferior frontal gyrus, and pars orbitalis (ORBinf.R and ORBinf.L). Weakened brain regions were mainly located in the occipital cortex, including the left calcarine sulcus (CAL.L), left middle occipital gyrus (MOG.L), parietal cortex left postcentral gyrus (PoCG.L), and right inferior parietal lobule (IPL.R).

**Figure 7:**
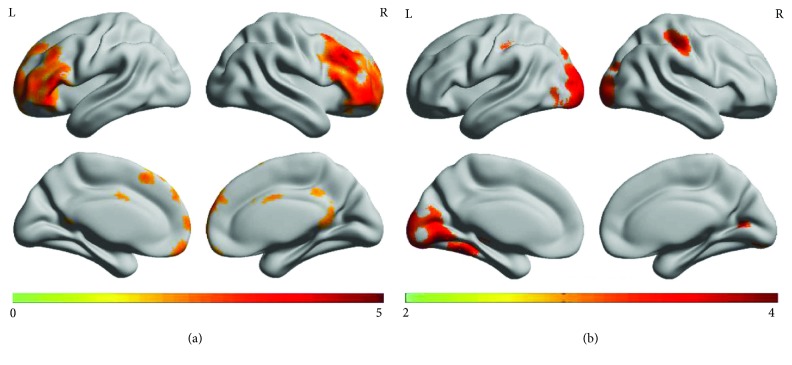
*Z*-statistics map. (a) *Z*-statistics map showing the brain regions that had strengthened connection with the IFGoperc.R region in the DLB group when compared to the HC group (*p* < 0.05, FDR corrected); (b) *Z*-statistics map showing the brain regions that had weakened connection with the IFGoperc.R region in the DLB group when compared to the HC group (*p* < 0.1, FDR corrected);

In this study, the DLB group was used as a reference for further analysis. *Z*-statistical mapping was performed in the IFGoperc.R region of the brain of the HC group.  Figure 7 illustrates the results obtained by *Z*-statistical test (*z*-map).

Compared to the HC group, the results of *Z*-statistics indicated brain regions that had strengthened connections with IFGoperc.R in the DLB group. These regions were located in the prefrontal cortex, which included the right middle frontal gyrus (MFG.R), bilateral inferior frontal gyrus, pars triangularis (IFGtriang.R and IFGtriang.L), right middle frontal gyrus, orbital part (ORBmid.R), and right medial frontal gyrus (SFGmed.R) (*p* < 0.05, FDR corrected).

## 4. Discussion

In this study, we found that the functional brain network of DLB patients was significantly different from that of healthy controls. Firstly, network alterations were broad in the DLB group. Secondly, divergent network alterations were manifested in the LDB group. Compared to the HC group, the DLB group suffered very severe dementia due to loss of characteristics of the small-world network. To perform seed correlation analysis, the brain region IFGoperc.R was selected as a seed in this study. Compared to the HC group, the results of *z*-map showed regions of strengthened connection with IFGoperc.R in the DLB group; these regions were located in the prefrontal cortex, and regions of weakened connection were located in the occipital cortex.

The physiological and pathological meanings of the above findings were discussed below.

### 4.1. Network Parameters

As shown in Figure 3, parameter *C* gradually becomes larger as the sparsity increases. When the HC group increases from 0.349 to 0.517, the DLB group increases from 0.305 to 0.668 over an entire threshold range. Statistical analysis based on permutation test showed that *C* was significantly higher in the DLB group than in the HC group at sparsity 10–14% and 21% (*p* < 0.05). Moreover, parameter *L* in the DLB group was significantly greater than that in the HC group when sparsity values were in the range of 21–40% (*p* < 0.05). At sparsity values of 31–40%, local E in the DLB group was significantly greater than that in the HC group. Global E in the DLB group was significantly lower than that in the HC group when sparsity values were in the range 7–22% (*p* < 0.05). Sigma in the DLB group was significantly smaller than that in the HC group when sparsity values were in the range 7–21% (*p* < 0.05). Finally, the local E values in the DLB group and HC group were approximately equal over the entire threshold range.

By accurately performing quantitative analysis, a small-world network was constructed. This network showed high global efficiency and optimal organizational structure, which better supported distributed information processing and extremely complex computation. In this study, small-world network was observed in both groups. The results of network parameters indicate that many changes were observed in the DLB group. Compared to the HC group, the characteristic path length was longer for the DLB group. Because characteristic path length was greater for observed points, the organization of the brain network was altered and network efficiency was lowered. In the DLB group brain network, the greater the clustering coefficient of the brain network, the longer would be the characteristic path length of the brain network in the DLB group. This network change tends to be a regular network, which has obvious shortcomings in communication and synchronization abilities of the signal when compared to a small-world network. Many diseases caused by disconnection of the nervous system were associated with loss of global characteristics of a small-world network, and they tend to form a regular network. A previous study had proved that in the DLB group [11], there was a significant loss in small-world network. This might be associated with presynaptic dysfunction, which is caused by *α*-synuclein aggregates present in the brain cortex, even at early stages of the disease. This finding was also confirmed in our research study.

### 4.2. Hubs of the Brain Network

Many studies have used fMRI and graph-based analysis method to investigate the functional network of the brain, revealing attributes such as small-world attributes [25, 26]. An important finding was that the networks governing the function of the human brain contained a small number of hubs, which were disproportionately associated with numerous connections. These hubs act as way stations for information processing by connecting distinct, functional specialized systems [27]. These brain hubs, which are mainly located in medial and lateral sections of frontal and parietal cortices, have higher rates of the following functions: cerebral blood flow, aerobic glycolysis, and oxidative glucose metabolism. They play vital roles in supporting fast communication across various brain regions [28]. In the present study, most brain hubs were located in the prefrontal and frontal cortices of the HC group. In the DLB group, brain hubs underwent significant changes, because normal hubs were attacked by dementia.

In present experiments, four significantly altered regions in the DLB groups were identified as hubs, including IFGoperc (IFG.R), olfactory cortex (OLF.R), hippocampus (HIP.R), and fusiform gyrus (FFG.L). This result could be proven in previous literature.

In a previous study conducted by Blanc et al., a significant variation in AAL brain regions was observed in the DLB group of patients. They found that in the DLB group, cortical thinning was found predominantly in the right temporoparietal junction, insula, cingulate, orbitofrontal, and lateral occipital cortices [29].

In a study conducted by Laura et al., significant changes were observed in the olfactory cortex and Lewy body pathology. In future studies, the metamorphosis of the olfactory cortex must be investigated to further elucidate the pathophysiology of DLB patients [30].

In DLB patients, major changes occur in the hippocampus of the human brain. Many studies have recorded the unique response of hippocampal CA2/3 in patients with Lewy body disease. By characterizing dystrophy in HIC hippocampal CA2/3 neurites, one may clarify how lesions lead to the development of dementia in DLB patients [31].

In a study conducted by Kosaka et al., a significant change was observed in the hippocampal gyri and fusiform gyri of a patient suffering from DLB. This disease was predominantly observed in the left region of the brain. This finding completely agrees with the conclusion of our current study [32, 33]. Thus, our experimental results have been validated with these findings.

### 4.3. Seed Correlation Analysis

The significantly altered region IFGoperc.R was selected as a seed. The results indicate that in the HC group, regions of strengthened connection were detected with IFGoperc.R seed. These regions were mainly located in the parietal cortex, including PoCG.R, PoCG.L, and MFG.R. Functional connection strength between prefrontal cortex and FGoperc.R seed was deep in the DLB group (its distribution was demarcated with a deep color.). In DLB patients, there were differences in the intrinsic functional connectivity of brains. Based on the differences in this connectivity, a new biomarker, IFGoperc.R, was obtained for precisely diagnosing DLB. In the DLB group of patients, there was also a significant decrease in the functional connectivity between extraordinary regions (LING.L, PoCG.R, SMG.R, and SOG.R) and IFGoperc.R seed. These findings were useful for neuroscientists to further understand the pathology of DLB.

### 4.4. Comparison between Chinese and Western Patients with DLB

Not many studies have investigated the parameters of the brain function network on DLB patients. For the first time, Peraza et al. conducted a representative study on the brain function network parameters [34] to understand the impact of fMRI in 22 DLB patients; however, this study was conducted on Western subjects with DLB. To further explore similar treatment processes for DLB patients in China, the relationship between DLB brain network changes and race was explored. By comparing experimental results with current results in Table 6, broadly similar results were obtained. However, the differences between Chinese and Western DLB people were as follows: larger *C* and *L* values and smaller values would be sigma and global efficiencies. This is an important point to consider because a change between these parameters was same as the difference between DLB and HC groups. To a certain extent, the parameter results of Chinese DLB patients were more serious than those of Western DLB patients. However, we cannot yet identify specific reasons for this difference. They also need to be studied further.

### 4.5. Limitations

In this study, there are still several limitations that need to be considered. Firstly, the gender distribution of the participants in the DLB group was extremely uneven (M : F = 21 : 1) in this study. The influences of gender for brain region analysis in the DLB group need be studied in the future. Secondly, the AAL template with 90 regions was used in this study. However, other templates with more regions could also be applied [35], and differences of brain region analysis amongst different templates need be studied. Thirdly, an unweighted and binary network was constructed in this study. Fourthly, partial correlation matrices were used to calculate network parameters and Pearson correlation was used for performing correlation analysis in this study. This may introduce bias in results. Differences of using various correlation matrices need be studied in the future.

## 5. Conclusion

By performing brain network analysis for FDG-PET images, this study systematically explored glucose metabolic brain network differences in HC and DLB groups in China. As a whole, a small-world topology was demonstrated by both groups. We found that the small-world network was severely impaired in the DLB group, implying that “small-world network” can be used as a biomarker for DLB. Based on seed ROI-based correlation analysis, the differences in brain functional connectivity were observed in DLB groups. One could obtain further insights into pathoetiological mechanisms of this condition. For better understanding and characterizing DLB patients and its triggering mechanisms, more relevant studies must be conducted. An accurate clinical diagnosis of DLB would then be possible.

## Figures and Tables

**Table 1:** Statistical information of all participants.

Info	HC (*n* = 22)	DLB (*n* = 22)	*p* value
Male : female	5 : 17	21 : 1	*p* < 0.001^a^
Age	63.5 ± 5.6	66.9 ± 8.4	*p* = 0.126^b^
MMSE	28.9 ± 1.3	20.0 ± 5.0	*p* < 0.001^b^

Age and MMSE are given as mean ± standard deviation. ^a^*χ*^2^ test, HC, and DLB. ^b^Analysis of variance, HC, and DLB.

**Table 2:** The definitions of network parameters used in this study.

Parameters	Abbreviation	Equations	Meaning
Clustering coefficient	*C*	C = (1/*N*)∑_*i*=1_^*N*^((2*E*(*i*))/(*k*_*i*_(*k*_*i*_ − 1))) *N*: number of nodes *K*_*i*_: number of nodes connected with node *i* *E*(*i*): actual connection edges among *k*_*i*_	A measure of the degree to nodes in a network which tend to cluster together
Characteristic path length	*L*	*L* = 1/((1/*N*(*N* − 1))(Σ_*i*,*j*∈*V*,*i*≠*j*_(1/*d*_*ij*_))) *N*: number of nodes in a network *d*_*ij*_: the shortest path between node *i* and j	A measure of the efficiency of the information or mass transport on a network

Gamma	Gamma	Gamma = *C*/*C*_rand_ *C*_rand_: clustering coefficient of the corresponding random network	A network could be defined as small-world network when gamma ≫ 1, lambda~1, and sigma > 1
Lambda	Lambda	Lambda = *L*/*L*_rand_ *L*_rand_: path length of the corresponding random network	
Small-world coefficient	Sigma	Sigma = gamma/lambda	

Global efficiency	Global E	Global E = (1/*N*(*N* − 1))∑_*i*,*j*∈*V*,*i*≠*j*_(1/*d*_*ij*_)	Measures how efficiently the network exchanges information
local efficiency	Local E	Local E = (1/*N*)∑_*k*∈*V*_((1/(*N*_*vk*_(*N*_*vk*_ − 1)))∑_*i*,*j*∈*V*,*i*≠*j*_(1/*d*_*ij*_))*N*_*vk*_: subgraph of node *k*	
Betweenness centrality	BC	BC_*k*_ = ∑_*i*,*j*∈*V*,*i*≠*j*_((*n*_*ij*_(*k*))/*n*_*ij*_) *n*_*ij*_(*k*): shortest path pass between nodes *i* and *j* through node *k*	An indicator of a node's centrality in a network

**Table 3:** The hub regions of the HC group.

Hubs	Anatomical classification	Bi (>1.5)	*p* with DLB value
*IFGoperc.R*	*Prefontal*	*1.83*	*0.0295* ^a^
SMA.L	Frontal	1.70	0.058
SFGmed.L	Prefontal	2.09	0.218
SFGmed.R	Prefontal	1.69	0.059
ORBsupmed.R	Prefontal	1.61	0.3265
INS.L	Subcortical	1.51	0.109
*INS.R*	*Subcortical*	*2.50*	*0.037* ^a^
DCG.R	Frontal	1.55	0.108
AMYG.L	Temporal	2.02	0.1035
CUN.L	Occipital	3.01	0.1065
*CUN.R*	*Occipital*	*2.62*	*0.041* ^a^
*LING.R*	*Occipital*	*1.59*	*0.135* ^a^
*PUT.R*	*Subcortical*	*2.71*	*0.138* ^a^
PAL.R	Subcortical	2.62	0.2475
*ITG.L*	*Temporal*	*1.70*	*0.0155* ^a^
*ITG.R*	*Temporal*	*2.18*	*0.0260* ^a^

Hub is defined as the brain region with a bi value > 1.5; class represents the functional classification of the corresponding brain regions; bi represents the normalized betweenness centrality; and the *p* with DLB value represents the statistical *p* value of the permutation test between the DLB and HC groups. ^a^Statistically significant difference (*p* < 0.05).

**Table 4:** The hub regions of DLB group.

Hubs	Anatomical classification	Bi (>1.5)	*p* value
ORBsup.L	Prefontal	1.51	0.2185
ORBmid.L	Prefontal	1.73	0.23
ORBinf.R	Prefontal	1.92	0.0955
ROL.L	Frontal	2.59	0.173
OLF.L	Prefontal	3.83	0.134
*OLF.R*	*Prefontal*	*4.02*	*0.015* ^a^
ORBsupmed.L	Prefontal	1.96	0.3045
REC.L	Prefontal	1.55	0.374
INS.L	Subcortical	3.67	0.109
*HIP.R*	*Temporal*	*5.19*	*0.008* ^a^
CUN.L	Occipital	1.63	0.1065
SOG.R	Occipital	2.23	0.1375
MOG.R	Occipital	2.69	0.115
IOG.L	Occipital	1.68	0.2235
IOG.R	Occipital	2.04	0.204
*FFG.L*	*Temporal*	*2.65*	*0.0135* ^a^
IPL.L	Parietal	2.26	0.128
ANG.R	Parietal	1.54	0.28
PCUN.L	Parietal	2.27	0.0895
PAL.R	Subcortical	1.82	0.2475
THA.R	Subcortical	3.88	0.0675

Hub is defined as the brain region with a bi value > 1.5; class represents the functional classification of the corresponding brain regions; bi represents the normalized betweenness centrality; and *p* value indicates statistic meaning. ^a^Statistically significant difference (*p* < 0.05).

**Table 5:** The significantly altered hubs in the DLB group compared to those in the HC group.

Hubs	Anatomical classification	Bi in HC	Bi in DLB	*p* value
IFGoperc.R	Prefontal	**1.83**	0.008	0.0295^a^
OLF.R	Prefontal	0.24	**4.02**	0.015^a^
HIP.R	Temporal	0.85	**5.19**	0.008^a^
FFG.L	Temporal	0.58	**2.65**	0.0135^a^

Compared with the HC group, the DLB group showed significantly different hubs. ^a^Statistically significant difference (*p* < 0.05).

**Table 6:** Comparison of the DLB network parameters between the present study and Western studies.

	Experimental image	*N*	*C*	*L*	Sigma	*E*	Gamma
Our study	FDG-PET	22	0.59	1.95	1.23	0.55	1.36
Western study [34]	fMRI	22	~0.52	~1.9	~1.35	~0.6	—

*N*: the number of subjects participating in the experiment.
